# A Correct FFR Trace Interpretation Is Important for a Clinical Decision

**DOI:** 10.1016/j.jaccas.2022.05.039

**Published:** 2022-10-19

**Authors:** Juan G. Chiabrando, Ignacio M. Seropian

We read with great interest the case report in by Bouaouina et al,^1^ who showed the successful occlusion of a coronary-to-pulmonary artery fistula after measuring functional significance by fractional flow reserve (FFR), and we congratulate them for the outcome.

As Bouaouina et al^1^ mentioned, coronary-to-pulmonary artery fistulas are infrequent and usually asymptomatic. However, an ischemia-inducing fistula may need surgical or percutaneous closure to treat symptoms.^2^

Bouaouina et al^1^ decided to perform FFR measurement under maximal hyperemia to unmask a steal phenomenon and eventually to occlude the fistula. They used an established cutoff threshold of FFR ≤0.8, which was exactly the result they reported. Although we agree with the proposed approach and cutoff threshold, we would like to caution about the proper interpretation of the FFR trace.^3^ FFR results from the relationship between the guide catheter mean pressure and the pressure wire mean pressure at the distal coronary artery. FFR measurement is performed on a beat-to-beat basis, with the lowest value provided by the FFR software. In Figure 1 of Bouaouina et al,^1^ it is represented as the purple line in the screen, with white horizontal lines representing the FFR scale from 0 to 1 (0.1 FFR per line). However, a closer look at the FFR tracing (Figure 1) shows that the value of FFR ≤0.8 provided may have been attributed to an artifact. In fact, the lowest FFR value shown in Figure 1 Bouaouina et al^1^ may be the result of an aberrant configuration of the pressure wave (second beat from left to right) that does not represent a normal pressure waveform. This is of the utmost importance for FFR interpretation,^3^^,^^4^ and it is usually not recognized by the FFR software. Many factors can alter the pressure form, including some involuntary manipulation of the guide catheter during measurement.^5^ Moreover, the purple line does not contact the 0.8 white line in the FFR, and thus the results provided by Bouaouina et al^1^ may not show any hemodynamically significant FFR value. Finally, most of the FFR values provided in Figure 1 before fistula occlusion have a value of 0.9 or above.

In summary, we would like to caution about a proper interpretation of an FFR measurement and advise using meticulous technique to improve outcomes.^6^
